# Acute-On-Chronic Mesenteric Ischemia: The Use of Fluorescence Guidance to Diagnose a Nonsurvivable Injury

**DOI:** 10.1155/2022/5459774

**Published:** 2022-02-07

**Authors:** Nova Szoka, Mathew Kahn

**Affiliations:** ^1^Department of Surgery, West Virginia University, 1 Medical Center Dr., Morgantown, WV 26505, USA; ^2^West Virginia University School of Medicine, USA

## Abstract

Mesenteric ischemia (MI) is a condition characterized by compromised intestinal perfusion, leading to varied patterns of bowel hypoxia that requires prompt diagnosis and surgical intervention. Here, we report a case in which indocyanine green (ICG) was utilized to evaluate intestinal blood flow in a patient with acute-on-chronic MI. A 65-year-old underweight female presented with abdominal pain out of proportion to exam and was found to have diffuse aortic atherosclerotic disease with chronic occlusion of both superior and inferior mesenteric arteries with distal reconstitution. After multidisciplinary evaluation, elective treatment with vascular surgery was planned; however, on day three of her hospitalization, the patient's abdominal pain acutely worsened. She was taken to the OR for exploratory laparotomy. Under white light, the small bowel from the ligament of Treitz (LOT) to the terminal ileum and the large bowel from the cecum to the splenic flexure appeared ischemic with patchy areas of necrosis. Fluorescence angiography was then performed; injection of indocyanine green (ICG) dye and imaging with the SPY-PHI near-infrared camera system demonstrated appropriate blood flow into the bowel mesentery, with complete absence of flow into the bowel mucosal surface from the LOT to the splenic flexure, confirming irreversible bowel necrosis. Introduction of ICG intraoperatively decreased the uncertainty associated with white light assessment of bowel viability, leading to a definitive intraoperative diagnosis and clear plan of care. The use of fluorescence guidance to diagnose fulminant small and large bowel necrosis prevented the surgical team from having to perform multiple takebacks to the operating room in the setting of a nonsurvivable injury. Had the surgical team relied on the white light appearance of the bowel, they would not have been able to diagnose the true extent of bowel demise. The patient was placed on comfort care for this devastating nonsurvivable injury.

## 1. Introduction

Despite being approved for medical uses in the 1950s, only in the past decade has the use of Indocyanine green (ICG) become commonplace in the field of general surgery. Its amphipathic properties allow it to bind lipoproteins and plasma proteins once injected into the blood stream or biliary system. Once distributed throughout the body ICG absorbs and emits light in the near-infrared range (750-800 nm) that penetrates deep into tissue and is detected by an imaging system [1–5]. The dye itself is processed in hepatocytes and excreted via bile with an average half-life of 3 minutes. Although this agent is contraindicated in those with iodine allergy, hypersensitivity reactions to ICG are exceedingly rare [1, 3, 5]. The fluorescence helps to identify the flow of blood and lymph in real time, providing surgeons with an augmented intraoperative reality and information that can improve clinical decision-making. Surgical techniques and procedures such as navigating around vital structures, tumor identification, lymph node dissection, cholecystectomies, and laparoscopic imaging can all benefit from the addition of intraoperative ICG [2, 3, 5]. The following case describes the use of ICG angiography in a patient with nonsurvivable acute-on-chronic mesenteric ischemia.

## 2. Case Presentation

A 65-year-old white female with past medical history of chronic obstructive pulmonary disease and a one-year history of chronic abdominal pain presented to the Emergency Department with a 2-day history of worsening epigastric pain. She had no prior abdominal surgeries. Her social history was significant for daily tobacco use. Despite having a normal appetite, the patient lost 30 pounds (27% of her total weight) over the past year. The patient's abdomen was soft and only mildly tender to epigastric palpation with negative rebound tenderness or guarding. Lab work demonstrated a normal white blood cell count and a normal lactate level. Abdominal CT angiogram revealed diffuse atherosclerotic processes, including a large mural thrombus within the aorta at the thoracolumbar junction, complete occlusion of the celiac trunk and superior mesenteric artery with distal reconstitution, and several splenic infarcts. The patient was admitted to the hospital and underwent evaluation by gastroenterology, general surgery, and vascular surgery. An upper endoscopy was performed to look for peptic ulcer disease; none was found. The care team concluded that symptoms were likely due to chronic mesenteric ischemia and the patient was scheduled for an open mesenteric bypass with vascular surgery, to be completed in several days.

**Figure 1:**
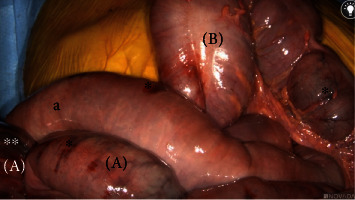
White light view of small intestines (a) and large intestines (b) with patchy ischemia (∗) and necrosis (∗∗) present.

**Figure 2:**
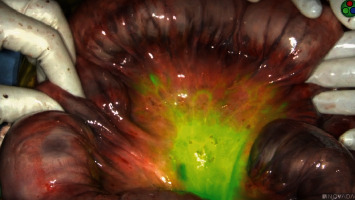
Indocyanine green perfusion to edge of small bowel mesentery but not beyond; no perfusion is present to the small bowel serosa.

**Figure 3:**
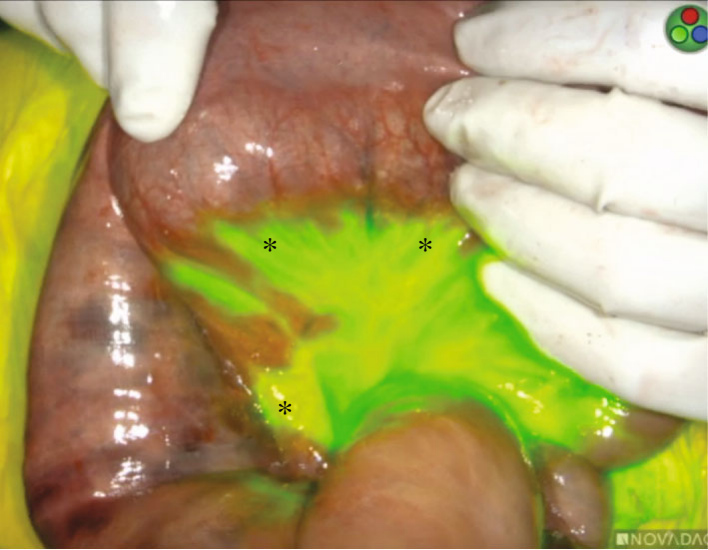
Perfusion to edge of small bowel mesentery but not beyond (∗); the small bowel serosa has no perfusion.

**Figure 4:**
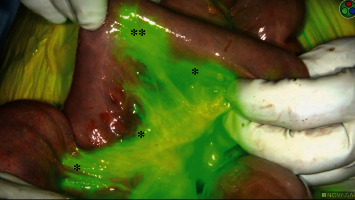
Perfusion to edge of small bowel mesentery but not beyond (∗) and one focal area of small bowel perfusion (∗∗).

**Figure 5:**
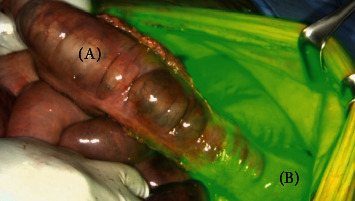
Nonperfused/ischemic colon (a) proximal to the splenic flexure and well-perfused colon (b) distal to splenic flexure.

**Figure 6:**
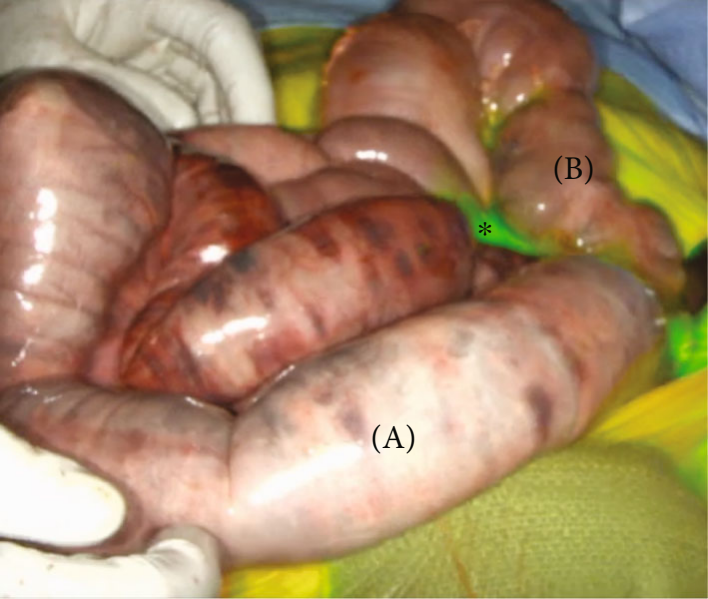
Perfusion to edge of mesentery but not beyond (∗) indicating nonperfusion to serosa and mucosa of the small intestines (a) and large intestines (b).

On hospital day five, the patient's abdominal pain acutely worsened in severity and lab work revealed leukocytosis of 14,400/L and a lactate level of 6.8 mmol/L. An emergent exploratory laparotomy was performed. Under white light alone, the small bowel from the ligament of Treitz (LOT) to the terminal ileum and the large bowel from the cecum to the splenic flexure appeared ischemic with patchy areas of necrosis (Figure 1). To obtain more information about the viability of the patient's bowel indocyanine green (ICG), angiography was performed using the handheld Stryker SPY-PHI system. Two milliliters of intravenous ICG was administered to evaluate vascular perfusion to the small and large bowel. The fluorescence given off from ICG could be easily visualized perfusing mesenteric vessels, but perfusion stopped at the edge of the mesentery and did not extend into the bowel wall of the entire small intestine (Figures 2–4) and the large intestine from the cecum to the splenic flexure (Figure 5). Lack of perfusion into the bowel serosa indicated occlusion of microvasculature, nonperfusion of both serosa and mucosa, and irreversible bowel necrosis (Figure 6). The entire small bowel and length of the colon extending to the splenic flexure had suffered from an irreversible degree of necrosis. This was deemed a terminal injury, and the patient received comfort care until expiring several hours later.

## 3. Discussion

Mesenteric ischemia is a condition in which abdominal vasculature is compromised leading to some degree of intestinal hypoperfusion. Acute mesenteric ischemia (AMI) is the more malevolent form in which blood flow is so restricted that bowel inflammation and necrosis results [6, 7]. AMI can be further divided into variants classified based off the mechanism of injury including arterial embolism, arterial thrombosis, venous thrombosis, and nonocclusive mesenteric ischemia [7]. Symptomology varies from patient to patient but can include diffuse abdominal pain out of proportion of physical symptoms, abdominal tenderness, nausea, vomiting, and diarrhea. Although it is a relatively uncommon complication, incidence is increased in individuals with cardiac disease, diffuse atherosclerosis, inflammatory bowel disease, and those of the geriatric population [6–12].

Diagnosis is made based largely on clinical suspicion, results of CT angiography, and visual inspection of the bowel via laparotomy or laparoscopy. Early detection is vital, as bowel that has not undergone extensive necrosis can be saved through revascularization techniques, increasing overall survival. If the bowel becomes necrotic, bowel resection is the only surgical option [6–8, 10, 12]. The mortality of acute mesenteric ischemia is variable and has been reported to be as high as 95% [10]. Chronic mesenteric ischemia (CMI) is a long-term complication resulting from partial occlusion of the mesenteric vasculature and diminished flow through the celiac trunk, SMA, or IMA. Patients suffering from CMI report chronic postprandial pain, weight loss despite adequate calorie intake, nausea, vomiting, and constipation. However, these symptoms usually do not manifest until at least two of the three major abdominal artery systems are affected [13]. Unlike AMI, chronic mesenteric ischemia is not a surgical emergency and can be corrected with revascularization procedures. As is sometimes the case, partial obstruction may continue to progress until there is complete occlusion of mesenteric vasculature, resulting in acute-on-chronic mesenteric ischemia. If this occurs, prompt surgical intervention is needed before bowel ischemia and necrosis occur. Patients who suffer from the arterial thrombotic variation of AMI often previously experienced symptoms of CMI for several months prior [6, 7, 9].

The high mortality rate of AMI can be partly attributed to the anastomotic network formed between the celiac trunk, SMA, and IMA. This interconnection helps minimize damage in situations where there is reduced flow through one of these major branches. However, when major occlusions do occur, it introduces a large degree of variation in the pattern of intestinal hypoperfusion that is produced [5]. Therefore, many patients initially present with atypical symptoms that do not fit the profile of AMI [7, 8, 14]. These factors are a dangerous combination for a condition in which early intervention is crucial and diagnosis relies heavily on clinical suspicion [6, 10]. Frequently, AMI is not diagnosed until patients are past the point where they can receive an angiography, as a result an exploratory laparotomy must be performed [6]. During this procedure, physicians must assess the viability of the entire bowel, determine if revascularization would be beneficial, and establish which sections of the intestine need to be resected. This is a difficult task under white light alone, as evaluating perfusion to the bowel mucosa can be challenging, in addition to each patient having a unique pattern of ischemia and necrosis [8]. The introduction of ICG during the evaluation of bowel perfusion has markedly improved the surgeon's ability to identify areas of bowel ischemia. Within thirty seconds of injecting ICG, the surgeon gains real-time visualization of which portions of the intestine are receiving an adequate blood supply [2, 3, 5]. This augmented surgical reality provided by fluorescence guidance gives the surgeon more precise information regarding the extent of bowel necrosis and where bowel resection should take place if needed.

In the case above, the patient initially presented to the emergency department with indicators of CMI. Despite having definitive CT angiography results, her transition towards AMI was not readily apparent. On hospital day 5, an acute change occurred, shifting her disease process to acute-on-chronic mesenteric ischemia. During exploratory laparotomy, using white light visualization, the degree of necrosis of her small and large intestine was not readily apparent. Under white light alone, the small bowel from the ligament of Treitz (LOT) to the terminal ileum and the large bowel from the cecum to the splenic flexure appeared ischemic with patchy areas of necrosis; however, some areas appeared to be viable. The use of ICG (Figures 1–6) demonstrated that perfusion stopped at the edge of the mesentery and did not extend into the bowel wall throughout the entire small bowel and majority of the large bowel.

The application of ICG provided several immediate benefits. The contrast between the dye and surrounding tissue provided clear and almost instantaneous results, confirming that irreversible bowel necrosis had already occurred. The use of fluorescence guidance increased the surgical team's certainty in declaring the patient had suffered from a nonsurvivable injury and eliminated the need for an interval second-look laparotomy. Although not a pleasant decision by any means, using fluorescence guidance to confirm of the extent of bowel necrosis spared the patient from any futile trips to the operating room.

## 4. Conclusion

Acute mesenteric ischemia (AMI) is a devastating condition in which prompt surgical intervention is crucial to the wellbeing of the patient. Unfortunately, the unpredictability of patient symptoms and the lack of a distinct diagnostic test makes recognition of the disease difficult. This uncertainty often follows the patient into the OR as surgeons must quickly determine the extent of bowel ischemia, which is difficult to see under white light alone. This case demonstrates how indocyanine green (ICG) and fluorescence guidance enabled the surgical team to diagnose a devastating bowel injury and minimize additional futile operative interventions.
